# Supplementary material for: Single Cell Transcriptomic and Chromatin Profiles Suggest Layer Vb Is the Only Layer With Shared Excitatory Cell Types in the Medial and Lateral Entorhinal Cortex
Source: Front Neural Circuits. 2022 Jan 26;15:806154. doi: 10.3389/fncir.2021.806154 (PMC8826650; doi:10.3389/fncir.2021.806154)
Supplement: Supplementary file 14 [file Data_Sheet_1.ZIP › MEC_Sup_vs_LEC_Sup_Motifs/homerResults/motif10.similar.html]

motif10

## Information for motif10

T
A
C
G
G
T
A
C
C
G
A
T
C
T
A
G
A
C
T
G
G
A
T
C
G
A
T
C
C
T
G
A
  
Reverse Opposite:  

G
A
C
T
C
T
A
G
C
T
A
G
T
G
A
C
G
A
T
C
G
C
T
A
A
C
T
G
A
T
G
C
  

|  |  |
| --- | --- |
| p-value: | 1e-55 |
| log p-value: | -1.270e+02 |
| Information Content per bp: | 1.801 |
| Number of Target Sequences with motif | 3759.0 |
| Percentage of Target Sequences with motif | 17.84% |
| Number of Background Sequences with motif | 1703.6 |
| Percentage of Background Sequences with motif | 13.96% |
| Average Position of motif in Targets | 346.5 +/- 259.5bp |
| Average Position of motif in Background | 335.7 +/- 272.2bp |
| Strand Bias (log2 ratio + to - strand density) | 0.0 |
| Multiplicity (# of sites on avg that occur together) | 1.12 |
| Motif File: | file (matrix) reverse opposite |

### Similar de novo motifs found

|  |  |  |  |  |  |  |  |
| --- | --- | --- | --- | --- | --- | --- | --- |
| Rank | Match Score | Redundant Motif | P-value | log P-value | % of Targets | % of Background | Motif file |
| 1 | 0.609 | A C G T A C T G A C T G A G T C A G T C A C T G C G T A C A G T | 1e-17 | -40.359278 | 1.07% | 0.57% | motif file (matrix) |
